# Three new country records from the genus *Limnephilus* Leach, 1815 (Trichoptera: Limnephilidae) from the Republic of Kosovo

**DOI:** 10.3897/BDJ.2.e4140

**Published:** 2014-11-18

**Authors:** Halil Ibrahimi, Agim Gashi, Astrit Bilalli, Milaim Musliu, Linda Grapci Kotori, Ferdije Zhushi Etemi

**Affiliations:** † University of Prishtina "Hasan Prishtina", Faculty of Mathematics and Natural Sciences, Department of Biology, Prishtina, Kosovo; ‡ University of Prishtina, Faculty of Mathematics and Natural Sciences, Department of Biology, Prishtina, Kosovo; § University of Peja "Haxhi Zeka", Faculty of Agribusiness, Klinë, Kosovo

**Keywords:** Trichoptera, *Limnephilus bipunctatus*, *Limnephilus decipiens*, *Limnephilus stigma*, Kosovo

## Abstract

New faunistic data on Trichoptera from Kosovo based on sampling carried out during the autumn of 2013 and first half of 2014 are presented. *Limnephilus bipunctatus* was found in a small stream in Kaqandoll village located in northern Kosovo and in Shtuticë village located in central Kosovo. Two male specimens of *Limnephilus decipiens*  were found at Gurrat e Hasan Agës Springs and Bistrica e Lloqanit River, an alpine area in the Lloqan mountains, which belong to the Bjeshkët e Nemuna mountains. A single male specimen of *Limnephilus stigma* was found in Klinë, located in central Kosovo. All three species are rare in Kosovo. A preliminary checklist of eight species of *Limnephilus* from Kosovo is provided along with biogeographical and ecological notes. This paper is a further contribution to the faunistic list of Trichoptera of Kosovo, one of the least explored countries in Europe.

## Introduction

The family Limnephilidae is a large Integripalpian family of caddisflies (Trichoptera) with more than 1000 species worldwide (Wiggins 1996), where they are found in marshes, lakes, rivers and streams from low altitudes up to the alpine area (Schmid 1988). The family is believed to have originated in North America from where they spread out into Siberia and Europe (Ivanov and Sukatsheva 2002). Larvae of this family are important in nutrient and energy transport through aquatic ecosystems (Wiggins 1996). The European limnephilid fauna contains more than 300 species from nearly 50 genera (Malicky 2014). *Limnephilus* Leach, 1815 is one of the most speciose genera of caddisflies, with nearly 200 described species (Nozaki 1996, Schmid 1988, Holzenthal et al. 2007). In Europe, this genus is represented by 59 species (Malicky 2004). However, recent DNA analysis of the family Limnephilidae showed that only 57 species  of *Limnephilus* are recognized as *Limnephilus* *sensu stricto* (about 25% of *Limnephilus* *sensu lato*) (Vshivkova 2006).

Historically, the caddisfly fauna of the Republic of Kosovo has been only occasionally investigated (Pongrácz 1923, Marinković-Gospodnetić 1975, Marinković-Gospodnetić 1980, Malicky 1986, Malicky 1999). Several investigations have been carried out during the last decade (Ibrahimi 2007, Ibrahimi and Gashi 2008, Oláh 2010, Ibrahimi et al. 2012a, Ibrahimi et al. 2012b, Ibrahimi et al. 2013, Oláh et al. 2013a, Oláh et al. 2013b), but this order of aquatic insects is still poorly known compared to some other Southeastern European countries. This study presents collection data for three species of *Limnephilus* not previously reported from Kosovo.

## Materials and methods

**Figure 1.**
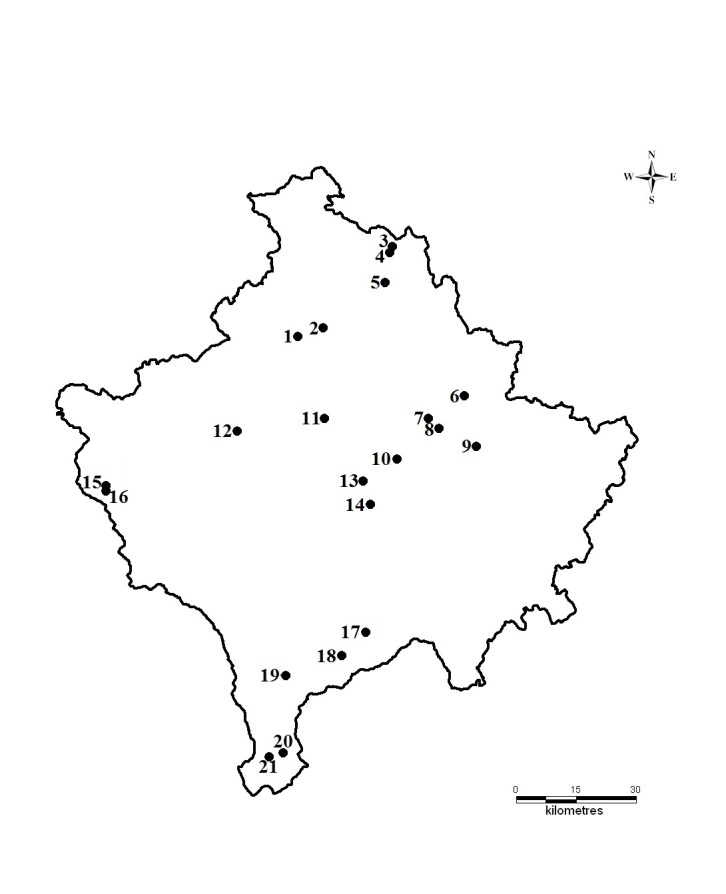
Localities sampled during 2013 and 2014 in Kosovo: 1. Ibër river, 2. Mazhiq stream, 3. Murgull river – Marincë, 4. Murgull river – Murgull, 5. Kaqandoll stream, 6. Siqevë stream, 7. Orllan stream, 8. Llukarë stream, 9. Marec stream, 10. Blinajë first lake, 11. Shtuticë, 12. Klinë, 13. Mollopolc stream, 14. Caralevë stream, 15. Lloqan river, 16. Te Gurrat e Hasan Agës stream, 17. Lepenc stream, 18. Lumbardhi i Pejës river – Prevallë, 19. Zaplluxhe stream, 20. Brod river, 21. Restelicë river.

Adult caddisflies were collected by using ultraviolet (UV) light traps, sweep nets, and casual handpicking close to the light sources. UV pyramid light traps were placed on stream banks and operated for approximately one hour and fifteen minutes after dusk. Sampling was carried out at 21 localities across Kosovo during the autumn of 2013 and spring and summer of 2014 (Fig. 1). Collected samples were preserved in 80% ethanol. The specimens were identified under a stereomicroscope using appropriate keys (Kumanski 1985, Kumanski 1988, Malicky 2004). Identifications were determined by the senior author. All specimens were identified to the species level with the exception of females of the genus *Tinodes* Curtis, 1834. The collection is deposited at the Department of Biology of the Faculty of Natural and Mathematical Sciences, University of Prishtina "Hasan Prishtina", Republic of Kosovo. Systematic presentation follows Morse (Morse 2014). ​

## Taxon treatments

### Limnephilus bipunctatus

Curtis, 1834

http://www.catalogueoflife.org/col/details/species/id/6873132

http://www.gbif.org/species/1442666

#### Materials

- **Type status:** Other material. **Occurrence:** recordedBy: Halil Ibrahimi; Fitesa Asllani Ibrahimi; Irsa Ibrahimi; Idlir Ibrahimi; individualCount: 2; sex: 1 male, 1 female; lifeStage: adult; **Taxon:** order: Trichoptera; family: Limnephilidae; genus: Limnephilus; specificEpithet: bipunctatus; **Location:** higherGeography: Europe; country: Kosovo; municipality: Mitrovicë; locality: Bajgorë area, entrance into the Kaçandoll village from Mitrovicë side; verbatimLocality: Sidespring of the Kaçandoll River by the main road; verbatimElevation: 1262 m; verbatimLatitude: 42.979°N; verbatimLongitude: 21.0509°E; **Event:** samplingProtocol: UV light trap; eventDate: 2013-09-25; fieldNotes: collected with ultraviolet light over the white pan operating from dusk until the next morning; eventRemarks: Other species associated with *Limnephilus bipunctatus* in this sample: *Potamophylax pallidus* (Klapalek, 1899) (2 males, 1 female), *Potamophylax cingulatus* (Stephens, 1837) (1 male, 3 females), *Wormaldia occipitalis* (Pictet, 1834) (1 male), *Chaetopteryx bosniaca* Marinkovic Gospodnetic, 1959 (2 males, 1 female); **Record Level:** institutionCode: University of Prishtina "Hasan Prishtina", Faculty of Mathematics and Natural Sciences, Department of Biology; collectionCode: caddisflies
- **Type status:** Other material. **Occurrence:** recordedBy: Astrit Bilallil; individualCount: 2; sex: 1 male, 1 female; lifeStage: adult; **Taxon:** order: Trichoptera; family: Limnephilidae; genus: Limnephilus; specificEpithet: bipunctatus; **Location:** higherGeography: Europe; country: Kosovo; municipality: Gllogoc; verbatimLocality: Shtuticë village, Bilallaj street; verbatimElevation: 740 m; verbatimLatitude: N42°41'53"; verbatimLongitude: E20°51'43"; **Event:** samplingProtocol: Normal light source; eventDate: 2013-09-29; fieldNotes: collected from the outside walls of the house close to the normal light source; eventRemarks: Other species associated with *Limnephilus bipunctatus* in these samples: 29.09.2013 *Micropterna nycterobia* McLachlan, 1875 (1 female); 03.10.2013 *Halesus digitatus* (von Paula Schrank, 1781) (1 male); **Record Level:** institutionCode: University of Prishtina "Hasan Prishtina", Faculty of Mathematics and Natural Sciences, Department of Biology; collectionCode: caddisflies

### Limnephilus decipiens

(Kolenati, 1848)

http://www.catalogueoflife.org/annual-checklist/2012/details/species/id/6896113

http://www.gbif.org/species/1442688

#### Materials

- **Type status:** Other material. **Occurrence:** recordedBy: Halil Ibrahimi; individualCount: 1; sex: male; **Taxon:** order: Trichoptera; family: Limnephilidae; genus: Limnephilus; specificEpithet: decipiens; **Location:** higherGeography: Europe; country: Kosovo; municipality: Deçan; locality: Bjeshkët e Nemuna Mountainous massive; verbatimLocality: Lloqan Mountains, Te Gurrat e Hasan Agës springs; verbatimElevation: 1991; verbatimLatitude: 42.557155°N; verbatimLongitude: 20.152696°E; **Event:** samplingProtocol: Entomological net; eventDate: 2014-08-12; fieldNotes: collected in the vegetation beside the stream; **Record Level:** institutionID: University of Prishtina "Hasan Prishtina, Faculty of Mathematics and Natural Sciences, Department of Biology; collectionID: caddisflies; institutionCode: University of Prishtina "Hasan Prishtina", Faculty of Mathematics and Natural Sciences, Department of Biology; collectionCode: caddisflies
- **Type status:** Other material. **Occurrence:** recordedBy: Halil Ibrahimi; Agim Gashi; Arif Kasumaj and Menderes Gashi; individualCount: 1; sex: male; **Taxon:** order: Trichoptera; family: Limnephilidae; genus: Limnephilus; specificEpithet: decipiens; **Location:** higherGeography: Europe; country: Kosovo; municipality: Deçan; locality: Bjeshkët e Nemuna Mountainous massive; verbatimLocality: Lloqan Mountains, Lumbardhi i Lloqanit River; verbatimElevation: 1666; verbatimLatitude: 42.5518°N; verbatimLongitude: 20.1624°E; **Event:** samplingProtocol: Entomological net; eventDate: 2014-08-12; fieldNotes: collected in the vegetation beside the river; eventRemarks: Other species associated with *Limnephilis decipiens* in this sample: *Rhyacophila tristis* Pictet, 1834 (4 males, 2 females), *Limnephilus auricula* Curtis, 1834 (6 males, 1 female), *Tinodes* sp. (1 female), Drusus cf. krusniki Malicky, 1981 (1 female); **Record Level:** institutionID: University of Prishtina "Hasan Prishtina, Faculty of Mathematics and Natural Sciences, Department of Biology; collectionID: caddisflies; institutionCode: University of Prishtina "Hasan Prishtina", Faculty of Mathematics and Natural Sciences, Department of Biology; collectionCode: caddisflies

### Limnephilus stigma

Curtis, 1834

http://www.catalogueoflife.org/annual-checklist/2008/show_species_details.php?record_id=784794

http://www.gbif.org/species/1442540

#### Materials

- **Type status:** Other material. **Occurrence:** recordedBy: Milaim Musliu; individualCount: 1; sex: male; **Taxon:** order: Trichoptera; family: Limnephilidae; genus: Limnephilus; specificEpithet: stigma; **Location:** higherGeography: Europe; country: Kosovo; county: Kosovo; municipality: Klinë; locality: town propper; verbatimLocality: 'Xhamia e Klinës" Mosque; verbatimElevation: 406; verbatimLatitude: 42.622631°N; verbatimLongitude: 20.575278°E; **Event:** samplingProtocol: by handpicking; eventDate: 2014-06-29; fieldNotes: collected from the inside walls of the mosque; **Record Level:** institutionCode: University of Prishtina "Hasan Prishtina", Faculty of Mathematics and Natural Sciences, Department of Biology; collectionCode: caddisflies

## Discussion

**Figure 2.**
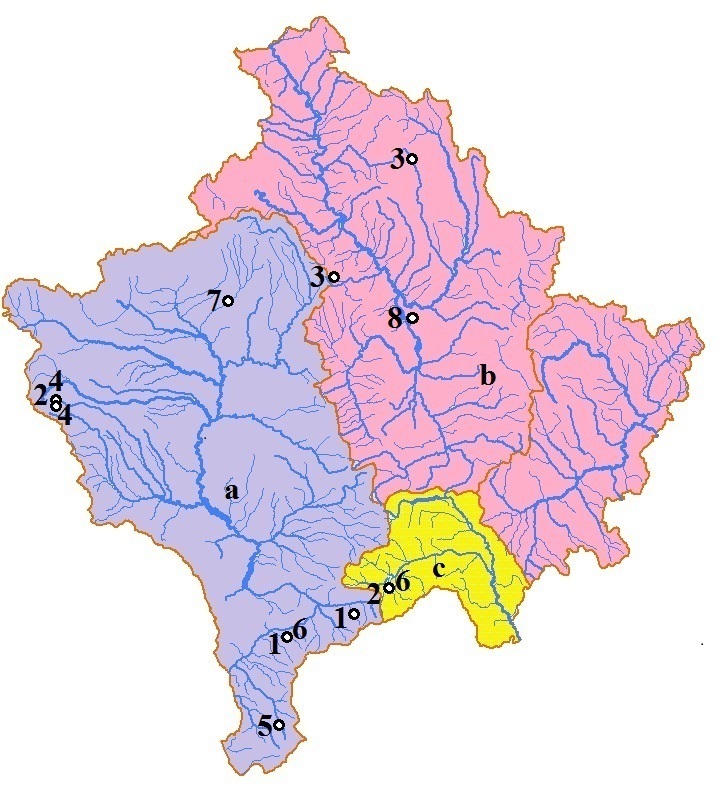
Distribution of *Limnephilus* species in Kosovo: 1. *Limnephilus affinis*, 2. *Limnephilus auricula*, 3. *Limnephilus bipunctatus*, 4. *Limnephilus decipiens*, 5. *Limnephilus petri*, 6. *Limnephilus sparsus*, 7. *Limnephilus stigma*, 8. *Limnephilus vittatus*. a - Adriatic Sea basin, b -  Black Sea basin, c - Aegean Sea basin.

*Limnephilis bipunctatus* is a typical inhabitant of small rivers and lakes, which can dry up in summer (Bouvet 1976, Wallace et al. 1990). Both sites where this species is found in Kosovo can exhibit considerably decreased water levels during summer. This species is notable for its extended flight period of up to six months and long imaginal diapause in the summer (Meyer and Mayer 2000). It is a widespread species from Europe and is also reported from the Balkan Peninsula, but it is not known to occur in Albania and Macedonia (Malicky 2014). Therefore, it's distributional range is considerably expanded by this study. The species seems to be rare in Kosovo. Out of more than 100 investigated localities in Kosovo during the last decade (Ibrahimi 2007, Oláh 2010, Ibrahimi et al. 2012a, Ibrahimi et al. 2012b, Ibrahimi et al. 2013, Oláh et al. 2013a, Oláh et al. 2013b) including spring areas, streams, rivers and at lesser degree lakes, ponds and marshes, this species was found only in two localities (Fig. 2). The Kaqandoll streamlet, one of the localities where this species was found during this investigation, has been intensively sampled previously (Ibrahimi et al. 2012a), but *Limnephilus bipunctatus* was not been found, suggesting that the abundance of the species in this locality may be extremely low.

*Limnephilus decipiens* is commonly found in other European countries where it occurs in lakes, marshes, and in midstream and downstream sections of rivers (Graf et al. 2008, Malicky 2014). In this study, it was found in a cold, fast flowing stream and river. *Limnephilus decipiens* has been previously collected in most of the countries neighboring Kosovo, but not in Albania and Serbia despite intensive collection efforts during the last decades (e.g. Marinković-Gospodnetić 1975, Marinković-Gospodnetić 1980, Živić et al. 2006, Oláh 2010, Oláh et al. 2013b). It's collection in Kosovo greatly expands it's known distribution. Currently, Gurrat e Hasan Agës Springs and upstream area of Bistrica e Lloqanit River are the only known localities where this species has been found in Kosovo (Fig. 2). Unlike its associated species *Limnephilus auricula*, which is abundant in this area, *Limnephilus decipiens* seems to be of considerably low abundance in this area.

*Limnephilus stigma* is present throughout central, western and northern Europe but apparently its distribution does not extend much towards the south (Malicky 2014). This species has also been reported from the Balkan Peninsula, but it has not been collected from Albania, Macedonia, Serbia and Montenegro. Monthly sampling during 2010 from the Klina River and Drini i Bardhë River did not yield any specimens of this species (Ibrahimi 2011). The location where this rare species was collected during this investigation is less than two kilometers from both sampling sites investigated during 2010 (Ibrahimi 2011).

Eight species of *Limnephilus* are now known from Kosovo (Table 1). Most of the species are restricted to localities in the Adriatic Sea Basin in Kosovo while fewer species are known from the Black Sea Basin and Aegean Sea Basin. All eight species of the genus *Limnehpilus* present in Kosovo are considered rare and always found in low abundance. Out of nearly 100 sampling stations in Kosovo (Ibrahimi 2007, Ibrahimi 2011, Ibrahimi and Gashi 2008, Ibrahimi et al. 2013, Ibrahimi et al. 2012a, Ibrahimi et al. 2012b, Oláh et al. 2013a, Oláh 2010, Oláh et al. 2013b), including those of this study, all of the species of *Limnephilus* are found either in one or two locations. The number of species of *Limnephilus* occurring in Kosovo is expected to increase with additional sampling of lentic habitats. Most of the currently sampled stations in Kosovo are springs, small streams, larger streams and rivers while lakes, ponds and marshes have been sampled more sparsely.

## Supplementary Material

XML Treatment for Limnephilus bipunctatus

XML Treatment for Limnephilus decipiens

XML Treatment for Limnephilus stigma

## Figures and Tables

**Table 1.** Checklist of the genus *Limnephilus* species in Kosovo with distributional, habitat and occurrence characteristics.

	Species	Adriatic Sea Basin	Black Sea Basin	Aegean Sea Basin	Total localities found in Kosovo	Habitats
1	*Limnephilus affinis* Curtis, 1834	+			2	lotic
2	*Limnephilus auricula* Curtis, 1834	+		+	2	lotic
3	*Limnephilus bipunctatus* Curtis, 1834	+	+		2	lotic
4	*Limnephilus decipiens* (Kolenati, 1848)	+			2	lotic
5	*Limnephilus petri* Marinković Gospodnetić, 1966	+			1	lotic
6	*Limnephilus sparsus* Curtis, 1834	+		+	2	lotic
7	*Limnephilus stigma* Curtis, 1834	+			1	lotic
8	*Limnephilus vittatus* (Fabricius, 1798)		+		1	lentic
